# Metagenomic insights into jellyfish-associated microbiome dynamics during strobilation

**DOI:** 10.1093/ismeco/ycae036

**Published:** 2024-03-15

**Authors:** Saijun Peng, Lijing Ye, Yongxue Li, Fanghan Wang, Tingting Sun, Lei Wang, Jianmin Zhao, Zhijun Dong

**Affiliations:** Muping Coastal Environment Research Station, Yantai Institute of Coastal Zone Research, Chinese Academy of Sciences, Yantai, Shandong 264003, China; University of Chinese Academy of Sciences, Beijing 100049, China; Muping Coastal Environment Research Station, Yantai Institute of Coastal Zone Research, Chinese Academy of Sciences, Yantai, Shandong 264003, China; Muping Coastal Environment Research Station, Yantai Institute of Coastal Zone Research, Chinese Academy of Sciences, Yantai, Shandong 264003, China; University of Chinese Academy of Sciences, Beijing 100049, China; Muping Coastal Environment Research Station, Yantai Institute of Coastal Zone Research, Chinese Academy of Sciences, Yantai, Shandong 264003, China; University of Chinese Academy of Sciences, Beijing 100049, China; Muping Coastal Environment Research Station, Yantai Institute of Coastal Zone Research, Chinese Academy of Sciences, Yantai, Shandong 264003, China; Muping Coastal Environment Research Station, Yantai Institute of Coastal Zone Research, Chinese Academy of Sciences, Yantai, Shandong 264003, China; Muping Coastal Environment Research Station, Yantai Institute of Coastal Zone Research, Chinese Academy of Sciences, Yantai, Shandong 264003, China; University of Chinese Academy of Sciences, Beijing 100049, China; Muping Coastal Environment Research Station, Yantai Institute of Coastal Zone Research, Chinese Academy of Sciences, Yantai, Shandong 264003, China; University of Chinese Academy of Sciences, Beijing 100049, China

**Keywords:** metamorphosis, microbiome, metagenome, symbioses, jellyfish blooms

## Abstract

Host-associated microbiomes can play key roles in the metamorphosis of animals. Most scyphozoan jellyfish undergo strobilation in their life cycles, similar to metamorphosis in classic bilaterians. The exploration of jellyfish microbiomes may elucidate the ancestral mechanisms and evolutionary trajectories of metazoan–microbe associations and interactions during metamorphosis. However, current knowledge of the functional features of jellyfish microbiomes remains limited. Here, we performed a genome-centric analysis of associated microbiota across four successive life stages (polyp, early strobila, advanced strobila, and ephyra) during strobilation in the common jellyfish *Aurelia coerulea*. We observed shifts in taxonomic and functional diversity of microbiomes across distinct stages and proposed that the low microbial diversity in ephyra stage may be correlated with the high expression of the host-derived antimicrobial peptide aurelin. Furthermore, we recovered 43 high-quality metagenome-assembled genomes and determined the nutritional potential of the dominant *Vibrio* members. Interestingly, we observed increased abundances of genes related to the biosynthesis of amino acids, vitamins, and cofactors, as well as carbon fixation during the loss of host feeding ability, indicating the functional potential of *Aurelia*-associated microbiota to support the synthesis of essential nutrients. We also identified several potential mechanisms by which jellyfish-associated microbes establish stage-specific community structures and maintain stable colonization in dynamic host environments, including eukaryotic-like protein production, bacterial secretion systems, restriction-modification systems, and clustered regularly interspaced short palindromic repeats-Cas systems. Our study characterizes unique taxonomic and functional changes in jellyfish microbiomes during strobilation and provides foundations for uncovering the ancestral mechanism of host–microbe interactions during metamorphosis.

## Introduction

Associations between multicellular organisms and microbes are widespread in nature and represent a driving force of evolution [1]. The rapid development and application of molecular sequencing technologies allowed biologists to recognize the diversity and ubiquity of microbial world and the complex communication between multicellular organisms and their associated microbes [2]. Accumulating evidence suggests that microbial symbionts participate in numerous host biological processes such as growth, feeding, digestion, immunity, adaptation, and detoxification [3–6]. Simultaneously, hosts can affect the community assembly process of symbiotic microbiota via various mechanisms, for example, variations in gene expression and differential adhesion to mucosal surfaces [7–9].

Recent studies have found microbial reorganization across various developmental stages of animal lifespan [10–14]. How these microbial associations are modulated to adapt to host dynamic selection throughout the life cycle is a fundamental question in interpreting many important biological processes from a “holobiont” perspective, particularly in organisms that undergo complex metamorphosis during development. Classic model organisms with complex life cycles, primarily insects and amphibians, present multiple difficulties in the exploration of host–microorganism interactions owing to the complexity of their body structures and behaviours [1]. Therefore, a simpler basal organism may provide new opportunities for the discovery of ancestral principles of microbial associations during metamorphosis [15].

Cnidarians (including Anthozoa and Medusozoa), a sister group of bilaterians and one of the earliest diverging metazoan clades, have simple body plans but complex life cycles. Most scyphozoans (Cnidaria, Medusozoa) undergo two metamorphoses in their life cycles, namely, planula-to-polyp and polyp-to-jellyfish transitions; the latter, termed strobilation, shares some molecular principles of metamorphosis with those seen in insects and amphibians [16]. Moreover, the strobilation process that realizes an increase of population size and the expansion of ecological niche (i.e. lifestyle transformation from benthic to planktonic) is one of the factors driving diversification and prosperity of scyphozoans, which have been reported to form periodic blooms in many sea areas worldwide [17–19]. The life-stage-specific diversity of associated microbiota has been uncovered in scyphozoans *Aurelia aurita* [20], *Aurelia coerulea* [6, 21], *Cyanea lamarckii* [22], *Chrysaora plocamia* [23], and *Chrysaora hysoscella* [22]. However, the typical marker gene (e.g. 16S rRNA) amplicon sequencing applied in existing studies cannot offer phylogenetic resolution below the species level and fails to provide information on absolute abundance and functional annotation, hindering a comprehensive understanding of the host–microorganism system during strobilation [24]. Metagenomic sequencing is a valuable tool for overcoming these limitations and identifying dramatic changes in the taxonomic and potential functional diversity of microbial communities across host life-history processes [25]. Exclusively, a study conducted using a metagenomic large-insert fosmid library argued that host-derived quorum quenching proteins may shape the developmental-stage-specific microbiota associated with *A. aurita* and may be involved in pathogen defence [26]. However, many mechanisms of interaction between these basal metazoans and their associated microbes remain largely unknown and need to be further investigated.

**Figure 1:**
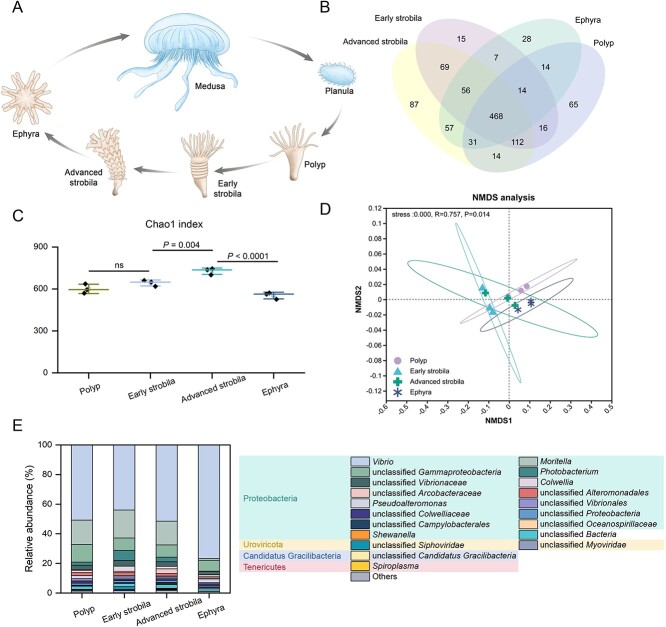
Microbiome diversity and composition during four stages of *A. coerulea* strobilation. (A) A schematic diagram of the life cycle of *A*. *coerulea*. (B) Statistics of shared and specific microbes across four life stages of *A*. *coerulea* at species level. (C) Alpha diversity of microbial communities across four life stages of *A*. *coerulea*. Data are represented as mean ± SD. Significant differences were determined by one-way ANOVA and Fisher’s LSD. (D) NMDS ordinations based on Bray–Curtis distance depicting the microbial community structures associated with four life stages of *A*. *coerulea*. Community dissimilarity was tested with an Adonis test. (E) Microbial community composition of four life stages of *A*. *coerulea*. Only top 20 genera are shown, and other low-abundance members are classified as “others.”

The widespread jellyfish *A. coerulea* is an ideal species for studying the relationship between metamorphosis and associated microbes. It has the unique advantages of a typical diphasic life cycle (sessile benthic phase and free-living pelagic phase), well-established laboratory culture methods, and easy access to samples. The necessity of robust microbial communities for the strobilation process has been confirmed in previous studies of the common moon jellyfish *A. aurita* and *A. coerulea*, with microbes functioning as promoters of strobilation initiation and regulators of host fitness [6, 27, 28]. In this study, a sterile culture environment was set up to eliminate the interference of opportunistic environmental microbes, making it more likely to reveal the core mechanism of microbial action in the ancient metamorphosis process. We used shotgun metagenomic sequencing to provide a detailed characterization of microbiome succession over four consecutive stages (polyp, early strobila, advanced strobila, and ephyra) during strobilation of *A. coerulea* (Fig. 1A). Our findings reveal the taxonomic and functional profiles of the *Aurelia* microbiome and highlight the potential roles of the associated microbes in nutrition and defence during *Aurelia* strobilation at the genomic level.

## Materials and methods

### Generation of sterile *Artemia* nauplii

The methods for generation of sterile *Artemia* nauplii are described by Peng *et al*. [6] with minor modifications. Briefly, *Artemia salina* cysts were hydrated at room temperature for 1 h and then bleached with sodium hypochlorite and NaOH solution to obtain naked cysts. Subsequently, the cysts were incubated in an oscillating incubator under light conditions at 27°C with shaking at 160 rpm for 24 h after removing the bleaching solution. Detailed steps are listed in the Supplementary Information. A lack of bacterial growth on 2216E plates after 3 days of incubation in 19°C and the absence of bands under 16S rRNA gene PCR amplification with primers 27F (5′-AGAGTTTGATCMTGGCTCAG-3′) and 1492R (5′-GGTTACCTTGTTACGACTT-3′) were considered confirmation of a sterile state.

### Sample collection, microbial enrichment, and community DNA extraction

*A. coerulea* polyps collected from the Yantai Haichang Whale Shark Ocean Park (Shandong, China) were kept in 1.5 L covered acrylic tanks under dark conditions in a 19°C incubator for a month to adapt to the laboratory environment. Subsequently, *A. coerulea* polyps were cooled from 19°C to 12°C to induce strobilation. During the adaptation and cooling induction periods, all animals were fed freshly hatched sterile *Artemia* nauplii in excess twice a week, and sterile artificial seawater was replaced 4 h after feeding. Monitoring was performed from the date of temperature reduction (Day 0), and the four stages of strobilation were sampled in a timely manner: polyp, early strobila, advanced strobila, and ephyra. *Aurelia* sampling from the four stages were completed until Day 28 with three replicates per stage and 30 individuals per replicate.

Microbial cells were enriched following established procedures [29] with minor modifications. The *Aurelia* samples were gently washed, homogenized, digested by collagenase III, centrifuged at 100 × *g* and filtered from tissue fragments. Detailed steps are described in the Supplementary Information. Then, the filtrate was centrifuged at 12 000 × *g* and 4°C for 20 min to precipitate microbial cells. Community DNA was extracted from the pellets using a DNeasy PowerSoil Kit (QIAGEN, Hilden, Germany), following the manufacturer’s instructions. The purity and concentration of extracted DNA were determined using a NanoDrop 2000 spectrophotometer (Thermo Fisher Scientific, Waltham, MA, USA).

### Metagenomic sequencing and metagenome assembly

Metagenome libraries were constructed using a NEXTflex™ Rapid DNA-Seq kit (Bioo Scientific, Austin, Texas, USA) according to the manufacturer’s instructions, with an insert size of 500 bp for each sample. Paired-end sequencing was performed on an Illumina NovaSeq 6000 platform (Illumina Inc., San Diego, CA, USA) by Majorbio Bio-Pharm Technology Co., Ltd (Shanghai, China). Raw reads from metagenomic sequencing were filtered to generate clean reads by removing low-quality reads with ambiguous N bases, length less than 50 bp, and quality score less than 20 using Fastp software (version 0.20.0, https://github.com/OpenGene/fastp). The clean reads were compared with the host genome sequences (NCBI accession number: PRJNA1005405) to remove host contamination using Bowtie2 software. The optimized reads were then assembled into contigs using MEGAHIT (version 1.2.9, https://github.com/voutcn/megahit), and the contigs with length ≥ 300 bp were selected as the final assembly results for downstream gene prediction.

### Gene prediction, taxonomic assignment, and functional annotation

The open reading frames (ORFs) of the assembled contigs were predicted using Prodigal (version 2.6.3, https://github.com/hyattpd/Prodigal). The predicted ORFs with length ≥ 100 bp were retrieved and translated into amino acid sequences. Non-redundant gene catalogues with identity ≥90% and coverage ≥90% were prepared using CD-HIT software (version 4.7, http://weizhongli-lab.org/cd-hit/). The high-quality reads were mapped to the non-redundant gene catalogues with identity ≥95% using SOAPaligner software (version soap2.21 release, http://soap.genomics.org.cn/), and gene abundance was evaluated by the number of reads [30]. The non-redundant gene catalogues were aligned to the Non-Redundant Protein Sequence (NR) database with an *e*-value cut-off of 1 × 10^−5^ using BLASTP (version 2.2.28+, http://blast.ncbi.nlm.nih.gov/Blast.cgi) for taxonomic assignment. Functional annotation was conducted by aligning the non-redundant gene catalogues to the Kyoto Encyclopedia of Genes and Genomes (KEGG) database and the Evolutionary Genealogy of Genes: Non-Supervised Orthologous Groups database with an *e*-value cut-off of 1 × 10^−5^ using BLASTP (version 2.2.28+).

The Chao1 index was calculated using Mothur software (version 1.30.1), and non-metric multidimensional scaling (NMDS) analysis was conducted based on Bray–Curtis distance using “vegan” package of R (version 4.3.2).

### Metagenome binning and phylogenetic analysis of metagenome-assembled genomes

Sequence splicing of the optimized reads was performed using MEGAHIT (version 1.2.9) with the following parameters: —min-contig-len 1000 —k-min 31. Genome bins were recovered using metagenomic binning software including MetaBAT2, Concoct, and Maxbin2 from the MetaWRAP pipeline (version 2.2.1) [31]. Non-redundant bins with completeness >50% and contamination <10% were obtained after merging and purification using RefineM (version 0.0.24, https://github.com/wwood/RefineM). The bins were dereplicated using dRep (version 3.4.2, https://github.com/MrOlm/drep) at the thresholds of -sa 0.99 and -nc 0.30 [32], and non-replicated metagenome-assembled genomes (MAGs) were generated. A CheckM workflow (version 1.0.12, https://github.com/Ecogenomics/CheckM/wiki) was used to estimate the completeness and contamination of these MAGs. Taxonomic classification of MAGs was performed using GTDB-TK software (version 1.7.0, https://github.com/Ecogenomics/GTDBTk). Subsequently, KEGG and Clusters of Orthologous Groups of Proteins (COG) database function annotations of the MAGs were performed using hmmscan and METABOLIC tools. A phylogenetic tree of the MAGs was constructed using Phylophlan software (version 2.1). Relative abundance of each MAG per sample was calculated using CoverM software (version 0.6.1, https://github.com/wwood/CoverM) by mapping the clean reads after quality control of the MAGs. The percentage of the total community captured by the MAGs was determined using SingleM software (version 0.12.1, https://github.com/wwood/singlem) by quantifying single copy marker genes in both the MAGs and the raw reads and calculating the fraction of marker genes recovered in the MAGs.

### Transcriptomic analysis of an antimicrobial peptide expression gene

We identified a gene that expresses an antimicrobial peptide (aurelin) from our previous RNA-seq data set representing the transcriptomes of four life stages (polyp, early strobila, advanced strobila, and ephyra) during the strobilation of *A. coerulea* (NCBI accession number: PRJNA1013748) [6]. This previous study and the present study used the same batch of polyps and set the same culture conditions; thus the transcriptomic results meet the premise of combined analysis with the microbiome data in this study. For technical details on the transcriptomic sequencing and differentially expressed gene analysis, see reference [6].

### Statistical analysis

Significant differences in the Chao1 index amongst the four stages were evaluated using one-way ANOVA and Fisher’s least significant difference (LSD) test. The generated Bray–Curtis dissimilarity matrix was tested using an Adonis method. Differences in the number of reads for KEGG modules, KEGG ontology (KO), and COG functions, as well as the relative abundance of MAGs between stages, were examined using Student’s *t* test.

## Results

### Overview of the microbial metagenome data set

A total of 80.4 Gb of sequencing information was obtained from 12 metagenomic samples. The average number of raw paired-end reads generated from shotgun sequencing was 92 499 345 per sample, and the average optimized reads obtained after removal of host contamination were 87 016 108 per sample (Table S1). The metagenomic assemblies from the 12 *Aurelia* samples yielded 88 869–138 873 contigs above 300 bp per sample (Table S2). Gene prediction produced 152 566–225 233 ORFs per sample with an average ORF length of 603.98–659.62 bp (Table S3). Taxonomic assignment against NR database identified three domains (Bacteria, Viruses, Archaea), 8 kingdoms, 58 phyla, 90 classes, 153 orders, 270 families, 487 genera, and 1053 species.

### Shifts in microbial community and functional diversity during metamorphosis

In total, 195 microbial species were stage specific to any of the four life stages of *A. coerulea*, accounting for 18.52% of the detected species (Fig. 1B). The microbial community richness of the advanced strobila stage was the highest amongst the four stages, whereas that of the ephyra stage was the lowest (Fig. 1C). NMDS analysis illustrated that the taxonomic composition of *Aurelia* microbiomes differed significantly across the four stages of strobilation (Adonis test, Fig. 1D). Interestingly, gene expression analysis of our previously published transcriptome data on the four life stages of *A. coerulea* strobilation [6] revealed differential expression of a known antimicrobial peptide, aurelin [33], with dramatic upregulation in the ephyra stage, suggesting a potential host-regulated pattern of low microbial richness in the *Aurelia* ephyrae (Fig. S1). Moreover, statistical analysis based on KO revealed that the α and β diversities of functional profiles of the microbial community varied distinctly across the four stages, indicating microbial functional reorganization during strobilation (Fig. S2).

The taxonomic classification showed that the *Aurelia* microbiome was dominated by Bacteria, followed by Viruses, and Archaea was rare. Prevailing phyla with relative abundance ≥1% were observed across all four life stages, amongst which bacterial phyla Proteobacteria, Candidatus Gracilibacteria and Tenericutes and viral phylum Uroviricota were the most abundant (Fig. 1E). In contrast, stage-specific phyla were all rare with low abundance (Table S4). *Vibrio*, *Moritella*, unclassified *Gammaproteobacteria*, *Photobacterium*, and unclassified *Vibrionaceae*, belonging to the bacterial phylum Proteobacteria, were the five most abundant genera associated with the four stages of *A. coerulea* (Fig. 1E). Comparative analysis of the adjacent stages of *A. coerulea* revealed a significant variance in the abundance of several *Vibrio* species between life stages, suggesting that the dominant *Vibrio* species may play an important role in various life stages (Student’s *t* test, Fig. S3).

Notably, the metagenomic assembly identified six viral phyla and seven archaeal phyla associated with *A. coerulea* (Figs S4 and S5). Based on KEGG pathway annotation, 13 potential functions of 17 *Aurelia*-associated viral families were identified, such as DNA replication, homologous recombination, and nucleotide excision repair (Fig. S4). The archaeal members were assigned to two-component system, ABC transporters, and tuberculosis, etc. (Fig. S5).

### Role of microbial associations in nutrient supply during the loss of host feeding

**Figure 2:**
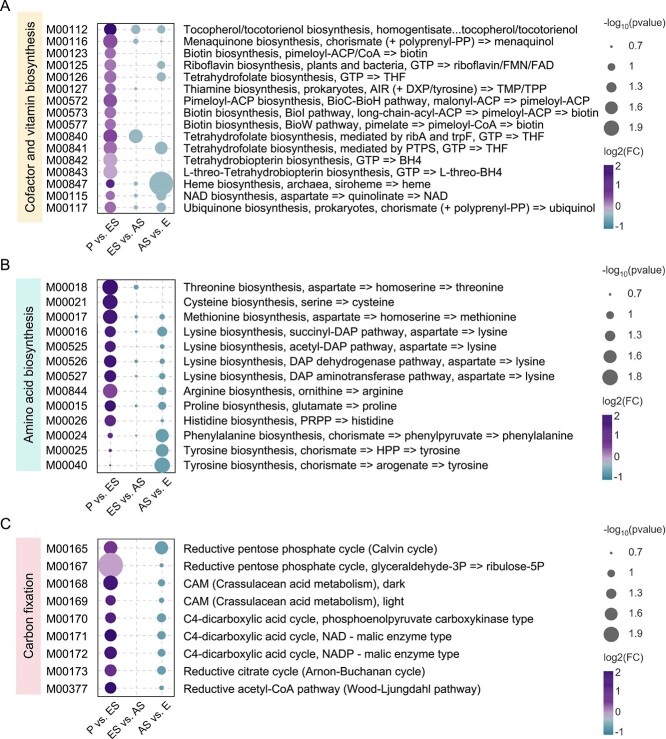
Nutrient biosynthesis functions of *Aurelia* microbiomes during four life stages. Bubble plots exhibit KEGG modules that were significantly different in the number of reads between adjacent stages of *A*. *coerulea*. (A–C) show the differential KEGG modules related to cofactor and vitamin biosynthesis, amino acid biosynthesis, and carbon fixation, respectively. Differences in the number of reads were analyzed by Student’s *t* test. P, polyp; ES, early strobila; AS, advanced strobila; E, ephyra.

The strobilation process of *Aurelia* is accompanied by a series of physiological changes: tentacles are reabsorbed, feeding behaviour of strobilae almost completely stops, transverse constrictions appear at the apical part of polyps, several disk-shaped segments are generated that subsequently turn into ephyrae (Fig. S6). The KEGG module analysis of the metagenome data set demonstrated the widespread potential of *Aurelia* microbiomes for the biosynthesis of essential nutrients for organismal metabolism, i.e. vitamins and their cofactors, amino acids, and fixed carbon (Table S5). The abundances of genes necessary to synthesize six vitamins (tocopherol, menaquinone, thiamine, riboflavin, biotin, and folate), five cofactors (tetrahydrobiopterin, L-threo-tetrahydrobiopterin, heme, NAD, and ubiquinone), and nine amino acids (threonine, cysteine, methionine, lysine, arginine, proline, histidine, phenylalanine, and tyrosine), as well as those corresponding to five carbon fixation types (the reductive pentose phosphate cycle, crassulacean acid metabolism, the C4-dicarboxylic acid cycle, the Arnon–Buchanan cycle, and the Wood–Ljungdahl pathway) were significantly higher in the two strobila stages than in the polyp and ephyra stages (Student’s *t* test, Fig. 2).

### Host–microbe recognition and antiviral defence strategies during metamorphosis

**Figure 3:**
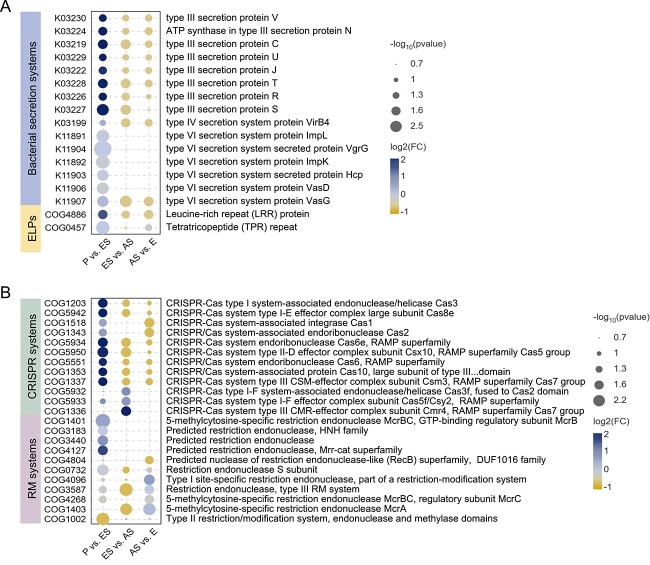
Functions linked with stable maintenance of *Aurelia* microbiomes during four life stages. (A) KOs involved in bacterial secretion systems and COGs belonging to eukaryotic-like proteins (ELPs) with stage-specific differences in the number of reads in *Aurelia* microbiomes. (B) COGs related to two antiviral systems that were significantly different in the number of reads between adjacent stages of *A*. *coerulea*. The statistical analysis method was Student’s *t* test. P, polyp; ES, early strobila; AS, advanced strobila; E, ephyra.

In the *Aurelia* strobilation process, the prevalence of genes linked with bacterial secretion systems and eukaryotic-like proteins (ELPs) potentially underpins host–microbe recognition and the stable establishment of microbial associations (Table S6, Fig. 3). The KOs of the Type III secretion system (T3SS) and Type IV secretion system (T4SS) associated with *A. coerulea*, which can inject effector proteins into adjacent host cells to modulate their metabolism [34], were significantly enriched in the early strobilae, whereas six KOs linked to the Type VI secretion system (T6SS) were enriched from the initiation of strobilation and remained abundant until the ephyra stage (Student’s *t* test, Fig. 3A). Second, ELPs identified in *Aurelia* microbiomes contained leucine-rich repeat (LRR), tetratricopeptide repeat (TPR), WD40 repeat, and ankyrin repeat proteins, in which the gene abundance of LRR and TPR proteins increased substantially in the early strobilae (Student’s *t* test, Table S6, Fig. 3A).

The gene abundance of multiple COGs related to the restriction-modification (RM) system and clustered regularly interspaced short palindromic repeats (CRISPR)-Cas system, two antiviral defence mechanisms of microbial communities, presented significant dissimilarity across the four life stages of *A. coerulea* (Student’s *t* test, Table S6, Fig. 3B). For instance, the gene abundances of five restriction endonuclease COGs were enriched in the early strobilae, whereas another two restriction endonuclease COGs (COG3440 and COG4804) were enriched in the advanced strobilae (Student’s *t* test, Table S6, Fig. 3B).

### MAGs and their functional profiles in *Aurelia* microbiomes

**Figure 4:**
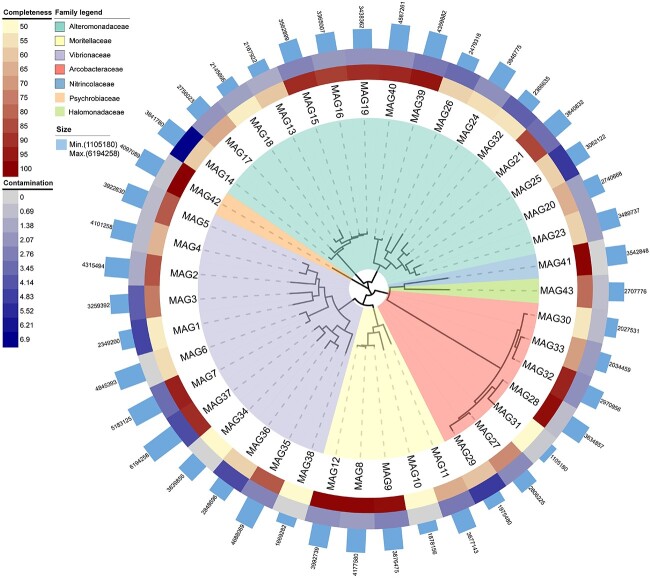
Phylogenetic tree of 43 MAGs recovered from *Aurelia* microbiomes. Concentric rings moving outward from the tree show completeness and contamination. The inferred families are marked on the tree with different colours. The bar plot of the outermost circle displays the size of each MAG.

A total of 43 MAGs (74.09% ± 17.48% completeness, 2.26% ± 1.62% contamination) were produced in 12 metagenomic biological samples from the four stages of *A. coerulea*, spanning bacterial phyla Proteobacteria and Campylobacterota (Table S7, Fig. 4). The recovered MAGs represented 38.40%–57.83% of the *A. coerulea* microbial communities, containing 10 genera (*Vibrio*, *Photobacterium*, *Moritella*, *Pseudoalteromonas*, *Cognaticolwellia*, *Colwellia*, *Neptuniibacter*, *Psychrobium*, *Oceanospirillum*, and *Poseidonibacter*) (Fig. S7A, Table S7). The taxonomic abundances of 24 recovered bacterial MAGs varied significantly between adjacent life stages (Student’s *t* test, Figs S7B and 5A). Importantly, *Cognaticolwellia* (one MAG), *Photobacterium* (two MAGs), and *Colwellia* (two MAGs) were statistically enriched in the early strobilae compared with the polyps, and the abundances of *Vibrio* (three MAGs), *Poseidonibacter* (six MAGs), and *Colwellia* (one MAG) decreased from the advanced strobilae to the ephyrae (Student’s *t* test, Figs S7B and 5A).

**Figure 5:**
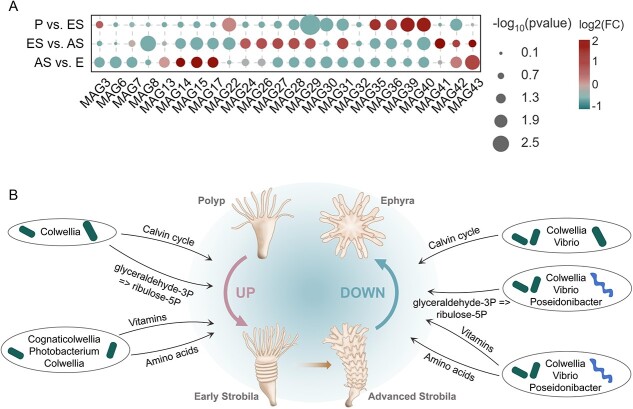
Stage-specific changes in the relative abundance and nutritional functions of MAGs. (A) Abundance patterns of 24 MAGs that varied significantly between adjacent life stages of *A. coerulea*. Statistical analysis was conducted by Student’s *t* test. (B) Schematic overview of nutritional contributions of MAGs with stage-specific differences in relative abundance. Only nutritional biosynthesis functions with upregulation from the polyp to early strobila stage and downregulation from the advanced strobila to ephyra stage are listed. P, polyp; ES, early strobila; AS, advanced strobila; E, ephyra.

Next, we determined the MAGs responsible for life-stage changes in the abundances of vitamin/amino acid biosynthesis and carbon fixation genes through functional profile analysis of the recovered microbial genomes (Fig. 5B). Among the 23 MAGs carrying genes essential for carbon fixation, two MAGs belonging to the genus *Colwellia* were potentially involved in providing more fixed carbon for the host in the strobila stages through two modules, the Calvin cycle and the conversion of glyceraldehyde-3P to ribulose-5P, whereas the low abundance of three MAGs from the genus *Vibrio*, MAG28 from the genus *Poseidonibacter*, and MAG40 from the genus *Colwellia* in the ephyrae may lead to less bacterial-derived fixed carbon production (Fig. 5B, Table S8). Five MAGs from genera *Cognaticolwellia* (MAG22), *Photobacterium* (MAG35, MAG36), and *Colwellia* (MAG39, MAG40) were significantly enriched upon the initiation of strobilation, triggering increases in gene abundance related to the biosynthesis of multiple vitamins (menaquinone, biotin, riboflavin, thiamine, and folate), cofactors (NAD and ubiquinone), and amino acids (lysine, methionine, threonine, proline, arginine, histidine, and cysteine) (Fig. 5B, Table S8). With the decay of several MAGs (MAG3, 6, 7, 27–30, 32, and 40) affiliated with the bacterial genera *Vibrio*, *Poseidonibacter*, and *Colwellia*, the abundance of genes encoding the above vitamin and amino acid biosynthesis decreased from the advanced strobilae to the ephyrae (Fig. 5B, Table S8).

All recovered MAGs possessed at least one of four eukaryotic-like domains (LRR, TPR, WD40 repeat, and ankyrin repeat) in their coding genes, reflecting a common feature of *Aurelia* microbiomes (Table S9). Five MAGs contained genes encoding eight KOs of the T3SS with significant difference between stages, including *Vibrio* sp. 013114595 (MAG6) and four MAGs from genus *Photobacterium* (Table S9). The life-stage-differential K03199 belonging to the T4SS was only encoded by MAG24 from the genus *Cognaticolwellia*, and six differential KOs associated with the T6SS were identified in 14 MAGs from the genera *Vibrio*, *Pseudoalteromonas*, *Photobacterium*, and *Oceanospirillum* (Table S9). The coding genes of life-stage-differential COGs linked with two antiviral defence mechanisms, the RM and CRISPR-Cas systems, were retrieved from 39 MAGs, not including *Vibrio tapetis* (MAG2), *Vibrio hepatarius* (MAG3), *Pseudoalteromonas undina* (MAG17), or *Photobacterium swingsii* (MAG38), manifesting a high gene incidence of these two defence mechanisms in *Aurelia* microbiomes (Table S9).

The high abundance and remarkable changes in members of the genus *Vibrio* over the four life stages of *A. coerulea* led to a strong interest in their functional profiles (Figs 1E and S3). We performed functional characterization of the seven MAGs from the genus *Vibrio* (Table S10). Genome binning revealed the functional potential of *Aurelia*-associated *Vibrio* in carbohydrate metabolism, carbon fixation, fatty acid biosynthesis and degradation, sulphur metabolism, nitrogen metabolism, etc., hinting an important role for *Vibrio* in nutrient flow and element cycling in the holobiont (Table S10).

## Discussion

Microbiomes, as integral parts of multicellular organisms, are closely related to a broad spectrum of host physiology and behaviour [35, 36]. Growing evidence indicates the involvement of microbes in the metamorphosis of animals with complex life histories [5, 28, 37]. The exploration of microbial associations in ancient jellyfish would provide new insights into the ancestral mechanisms of microbiome regulation during host metamorphosis. However, the function of jellyfish-associated microorganisms across different life stages remains poorly understood. Here, our comparative analyses of four life stages using shotgun metagenomic sequencing revealed intricate shifts in the taxonomic structures and functional potential of *Aurelia*-associated microbiota during strobilation that underpin the normal metamorphosis of the host.

Several studies have reported that the absence of polyp-associated microbes can cause some abnormal strobilation traits in *Aurelia*, but these abnormal traits are rescued by the recolonization of native microbiota of polyps [6, 27, 28]. These results indicated that certain microbes associated with *Aurelia* polyps play a key role in the regulation of host strobilation. However, the specific identity and functions of these microbes associated with *Aurelia* polyps are still largely unknown. Therefore, in the current study, sterile culture conditions were set up to exclude the interference of environmental opportunistic microorganisms and to explore the remodelling of the native microbiome of polyps and its potential functions in the strobilation process. In the present study, the strobilation process was successfully completed in the absence of environmental microorganisms, and the structure of the microbial community showed dramatic changes between host life stages.

Transcriptome comparison of the four stages of *Aurelia* revealed high expression of an *Aurelia*-specific antimicrobial peptide, aurelin, in the ephyra stage [33]. Coincidentally, microbial richness was lower in the ephyra stage than in the other three life stages. Several reports have confirmed that host-derived antimicrobial peptides shape both microbial community composition and abundance in *Hydra* [38, 39], *Drosophila* [40], scallops [41], and mice [42]. This evidence suggests a potential strategy for host-shaped distinct microbial communities over the life cycle other than quorum-quenching proteins in *Aurelia* [26]. However, a recent study indicated that bacterial colonization and succession may also be promoted by bacteria–bacteria interactions largely independent of the host ontogeny [43]. Thus, further experiments are necessary to determine the molecular mechanism of microbial succession during *Aurelia* strobilation.

We noted the dominance of *Vibrio* members in *A. coerulea* microbiomes at all sequenced stages. The prevalence of genus *Vibrio* in microbiomes has been identified in several jellyfish species, including *A. aurita* [20, 44, 45], *A. coerulea* [21, 46], *Nemopilema nomurai* [46], *Rhopilema esculentum* [46], *Rhizostoma pulmo* [47, 48], *Cotylorhiza tuberculata* [49], *C. lamarckii* [50], and *Cyanea capillata* [50, 51]. These results attract our focus to the functions of *Vibrio* as a core player in jellyfish–microbe associations. Based on KEGG module annotations, we displayed the genomic functional profiles of seven MAGs from the genus *Vibrio* in *Aurelia* associations and unearthed the active potential of these bacteria in the nutrient flow and element cycling (i.e. carbon, nitrogen, and sulphur) of the holobiont. Our study complements previous findings regarding the nutritional potential of *Vibrio* in addition to potential pathogens, further improving our understanding of *Vibrio* associations [47, 51].

Animals cannot synthesize certain vitamins and amino acids on their own and must acquire these essential nutrients from their diet or bacterial symbionts [52]. Genomic investigations of evolutionary ancient metazoans, namely, corals and sponges, supported that microbial partners are involved in nutrient acquisition for their eukaryotic hosts [53–55]. However, there is no genomic evidence of jellyfish microbiomes to determine whether their members also contribute nutritionally. Using metagenomic sequencing, the present study revealed a significant increase in the abundances of genes associated with the microbially mediated biosynthesis of amino acids, fixed carbon, vitamins, and cofactors essential for host development in the jellyfish fasting stages. This finding suggests that when jellyfish cannot obtain dietary nutrients, microbial community reorganization potentially supports more nutrient biosynthesis. The cessation of feeding behaviour during the strobila stages of jellyfish is analogous to the pupal stage of holometabolous insects. Similarly, the onsets of insect metamorphosis and of jellyfish strobilation both rely on the retinoid X receptor as a core element; that is, the two processes share similar molecular mechanisms [16]. Accordingly, research on holometabolous insects has shown that rebuilding an adult body benefits from nutrients and energy produced by symbiotic microbes [56–58]. We speculate that the provision of essential nutrients by associated microbes for the reconstruction of the host body plan during metamorphosis may be an ancestral mechanism of animals with complex life cycles.

In addition to studying their contribution to nutritional requirements, we investigated how the dynamic reorganization of microbial communities across distinct host life stages allows them to evade host immune phagocytosis and resist exogenous opportunistic infections. Functional gene profiling revealed a suite of genetic signatures in the jellyfish microbiome that may facilitate stable symbiotic associations. ELPs (LRR, TPR, WD40 repeat, and ankyrin repeat) with a high frequency in jellyfish microbiomes are common domains present in eukaryotes but are rarely encoded in bacterial and archaeal genomes [59]. However, recent studies have demonstrated that ELPs are widespread in coral and sponge microbiomes [53–55, 60, 61]. As indicators of a symbiotic lifestyle, these proteins mediate host–microbe recognition and protect microbial symbionts from phagocytosis by eukaryotic cells [62, 63]. Our study is the first to identify ELPs in jellyfish microbiomes, supporting the convergence of genetic characteristics of ancient metazoan symbionts.

To further understand host–microbe interactions during *A. coerulea* strobilation, we characterized the temporal dynamics of the three bacterial secretion systems across distinct jellyfish life stages, as secretion systems are thought to be closely related to successful microbial colonization in a variety of host systems, such as plants [64, 65], sponges [55], octocorals [60], and insects [66, 67]. We found that the abundances of T3SS and T4SS genes were elevated in the early strobila stage and were subsequently reduced, and those of T6SS genes were high from the early strobila stage to the ephyra stage. Concordantly, genes encoding the T3SS, which is responsible for the injection of bacterial effector molecules into eukaryotic cells, were observed to be upregulated during the early and mid-pupal stages in *Sitophilus* weevils [11, 68]. Moreover, the pioneering finding that an increase in T3SS-encoding gene expression underlies a transient “infectious state” of the endosymbiont during weevil metamorphosis was revealed [11]; surprisingly, this genetic feature is also present in the metamorphosis of basal metazoans. The enrichment of the T4SS has been reported in sponge symbionts, and this secretion system is believed to deliver ELPs to host cells [62, 69, 70]. The role of the T4SS in metamorphosis remains unclear. Considering the specificity of ELPs in ancient metazoan microbiomes, we propose that the involvement of the T4SS in metamorphosis might be a primitive mechanism that has been eliminated with evolution. The T6SS exhibited a different pattern of abundance dynamics from those of the T3SS and T4SS and showed a state of continuous enrichment after the initiation of *Aurelia* strobilation. The T6SS is regarded as an interbacterial competitive system of host-associated bacteria in response to shifts in physical or host environments [67, 71, 72]. Therefore, we speculate that the abundant T6SS-encoding genes may favour competitive colonization by bacteria during jellyfish strobilation. Moreover, the abundances of genes related to RM and CRISPR systems, as parts of the immune repertoire of bacteria and archaea, presented prevalence and dissimilarity during the strobilation of *A. coerulea*. Our metagenomic surveys recovered a diverse viral population from the jellyfish microbiomes. Effective defence mechanisms against viral infections via the identification and cleavage of foreign DNA are recognized as prerequisites for the survival of high cell-density prokaryotic consortia [60, 73–75].

In conclusion, the present study revealed that microbial community diversity and composition change significantly throughout the metamorphosis of *Aurelia*. We observed low microbial diversity in the ephyrae, which may be attributable to an antimicrobial peptide produced by *Aurelia*. We hypothesis that the dominant members of the genus *Vibrio* played a role in nutrient flow and element cycling during *Aurelia* strobilation. Comparative analysis based on gene abundance suggested that microbiota may help to compensate for host nutritional deficiency during the periods when the host stopped feeding and revealed the potential of four ELPs, three secretion systems, and two defence systems to facilitate specific and stable microbial colonization in distinct stages of *Aurelia* strobilation. However, we cannot deny that there are limitations in this study: (i) The microbial cell enrichment protocols, including collagenase digestion, filtration, and gradient centrifugation, which are still widely used in the study of marine animal metagenomics [29, 54, 73, 75], may cause the loss of certain microbial cells. (ii) Shotgun metagenomic sequencing provides more accurate absolute abundance and taxonomic classifications information than 16S rRNA gene sequencing; however, this methodology can only evaluate the functional potential at the genomic level, and can not directly represent the actual events. Future research should include detailed functional research using distinct technical methods, such as metatranscriptome sequencing and targeted culture of specific strains. Moreover, the impacts of microbial dynamics caused by complex environmental changes and biological contact from field environment on the metamorphosis of jellyfish should be investigated further.

## Supplementary Material

Supplementary_Information_ycae036

Supplementary_Tables_ycae036

Supplementary_Code_ycae036

## Data Availability

The shotgun metagenome sequences and the assembled MAG sequences obtained in this study have been deposited as a single project in the NCBI Sequence Read Archive under the accession number PRJNA1067395. The codes generated from this study are available in supplementary file.
