# Supplementary material for: Metagenomic insights into jellyfish-associated microbiome dynamics during strobilation
Source: ISME Commun. 2024 Mar 15;4(1):ycae036. doi: 10.1093/ismeco/ycae036 (PMC10988111; doi:10.1093/ismeco/ycae036)
Supplement: Supplementary_Information_ycae036 [file supplementary_information_ycae036.docx]

**Supplementary Materials and Methods**

**Generation of sterile *Artemia* nauplii**

*Artemia salina* cysts (~ 1 g) were hydrated in 90 ml sterile Milli-Q water at room temperature for 1 h. A mixed solution containing 50 mL sodium hypochlorite and 3.3 mL 32% NaOH solution was added to the hydrated cysts, gently shaken, and incubated for 6 min to obtain naked cysts. Then, 70 mL Na_2_S_2_O_3_ solution (10 mg/L) was added to terminate the reaction. Subsequently, the cysts were rinsed with 5 L sterile Milli-Q water in a 100 μm-sterile sieve to completely remove the bleaching solution. Finally, the treated cysts were transferred into 500 mL of sterile artificial seawater (ASW, prepared from Reef Crystals [Instant Ocean, Blacksburg, VA, USA] to a salinity of 30 ppt and then filtered through 0.22-µm filter membranes), covered with a sterile sealing film, and incubated in an oscillating incubator under light conditions at 27 °C and 160 rpm for 24 h.

**Microbial enrichment**

The *Aurelia* samples were gently washed three times in sterile calcium/magnesium-free seawater (CMFSW; 0.4 M NaCl, 10 mM KCl, 7 mM Na_2_SO_4_, and 0.05 mM NaHCO_3_ in Mill-Q water) to remove the loosely attached microbes on the surface and then homogenised with a T10 basic ULTRA-TURRAX homogeniser (IKA, Staufen, Germany) for approximately 20 s in 10 mL fresh sterile CMFSW. All the instruments and equipment were washed thoroughly with 70% ethanol and sterile CMFSW. Collagenase Ⅲ (ACMEC, Shanghai, China) was diluted with CMFSW, filtered through a 0.22-µm filter membrane and added to each sample to a concentration of 0.5 g/L. Then, samples were mixed and incubated at 19 ℃ for 4 h to release the embedded microbes. Each sample was filtered through a 100 μm-Nylon sterile cell strainer (BBI, Shanghai, China) and centrifuged for 15 min at 100 × *g* and 4 ℃ to remove the remaining jellyfish tissue and cell fragments. The supernatant was filtered twice through a 5-μm syringe-type microporous membrane filter, and then, the filtrate was centrifuged at 12000 × *g* and 4 ℃ for 20 min to precipitate microbial cells.

**Supplementary Figures**


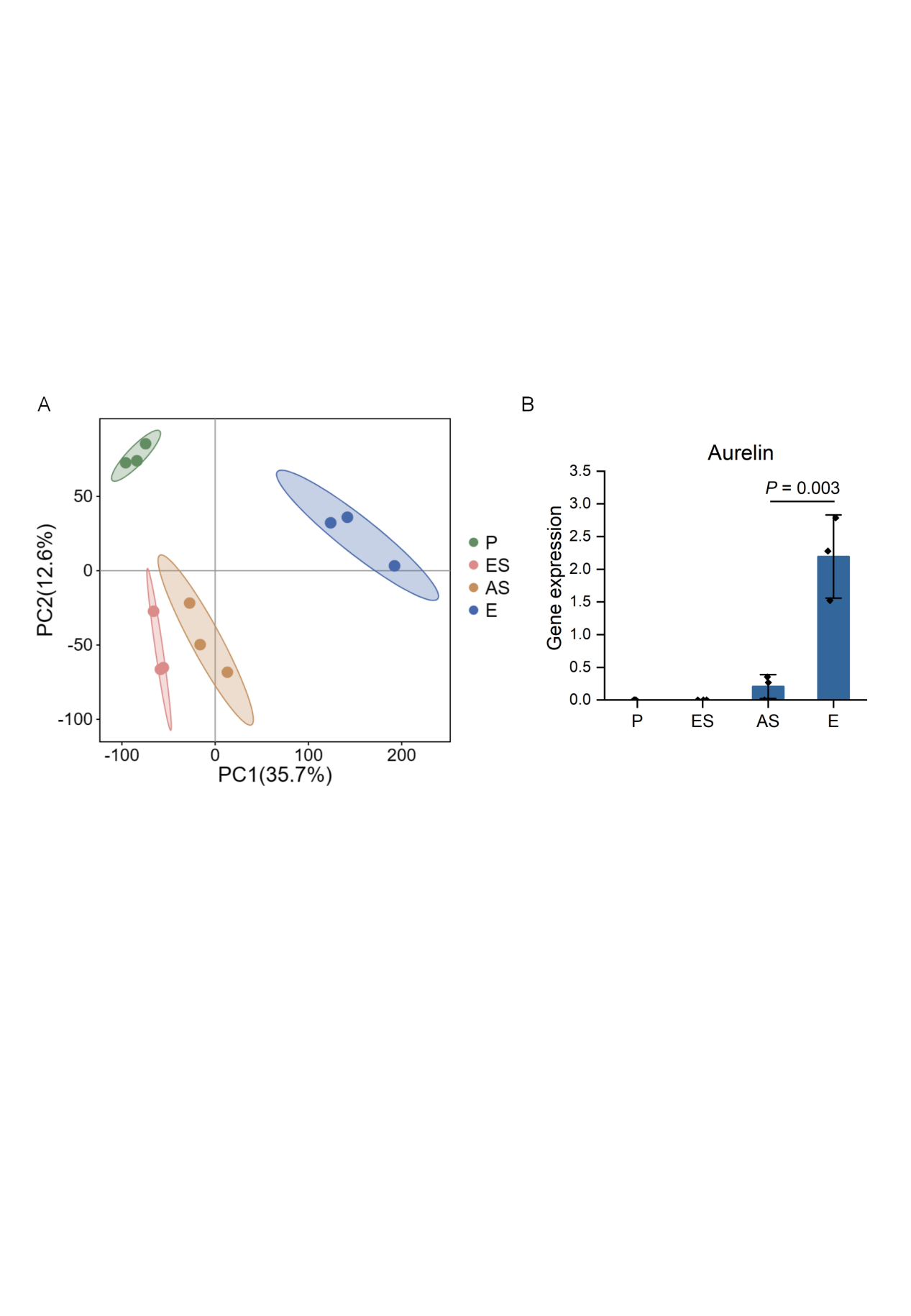


**Fig. S1 Transcriptome profiles and antimicrobial peptide (aurelin) gene expression patterns during four life stages of *Aurelia coerulea*.** (A) Principal component analysis (PCA) showing life-stage differences in gene expression patterns of *Aurelia*. (B) FPKM of differentially expressed gene for aurelin synthesis. The *P*-value refers to the corrected FDR value. Data are represented as mean ± SD. P, polyp; ES, early strobila; AS, advanced strobila; E, ephyra.


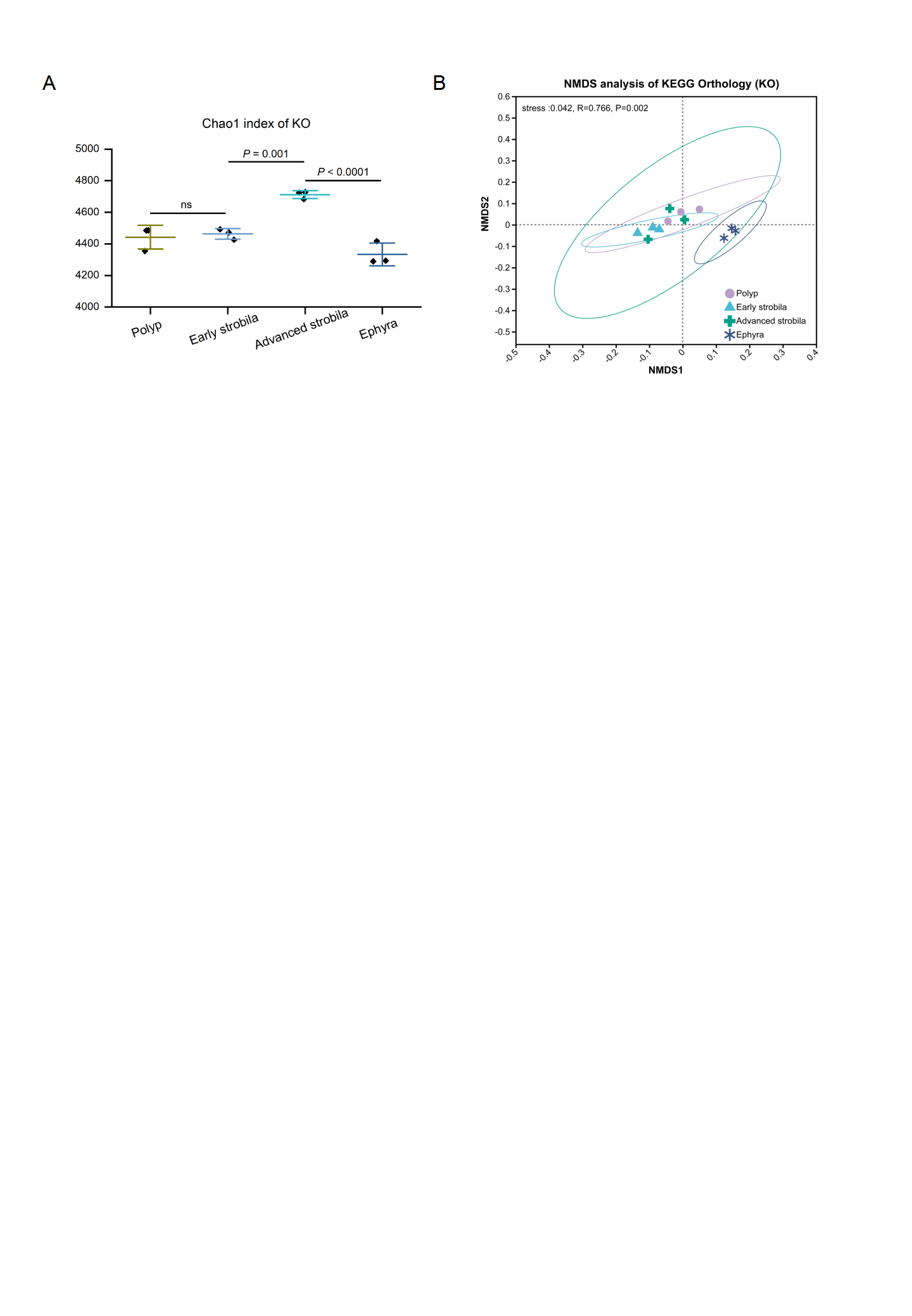


**Fig. S2 Functional diversity and structure of *Aurelia coerulea* microbiomes.** (A) KO functional diversity of microbiomes across four life stages of *A. coerulea*. Data are represented as mean ± SD. Significant differences were determined by one-way ANOVA and Fisher’s LSD. (B) Nonmetric multidimensional scaling (NMDS) ordinations based on Bray–Curtis distance of KO to assess functional reconstruction of microbiomes across four life stages of *A. coerulea*. Community dissimilarity was tested with an adonis test.


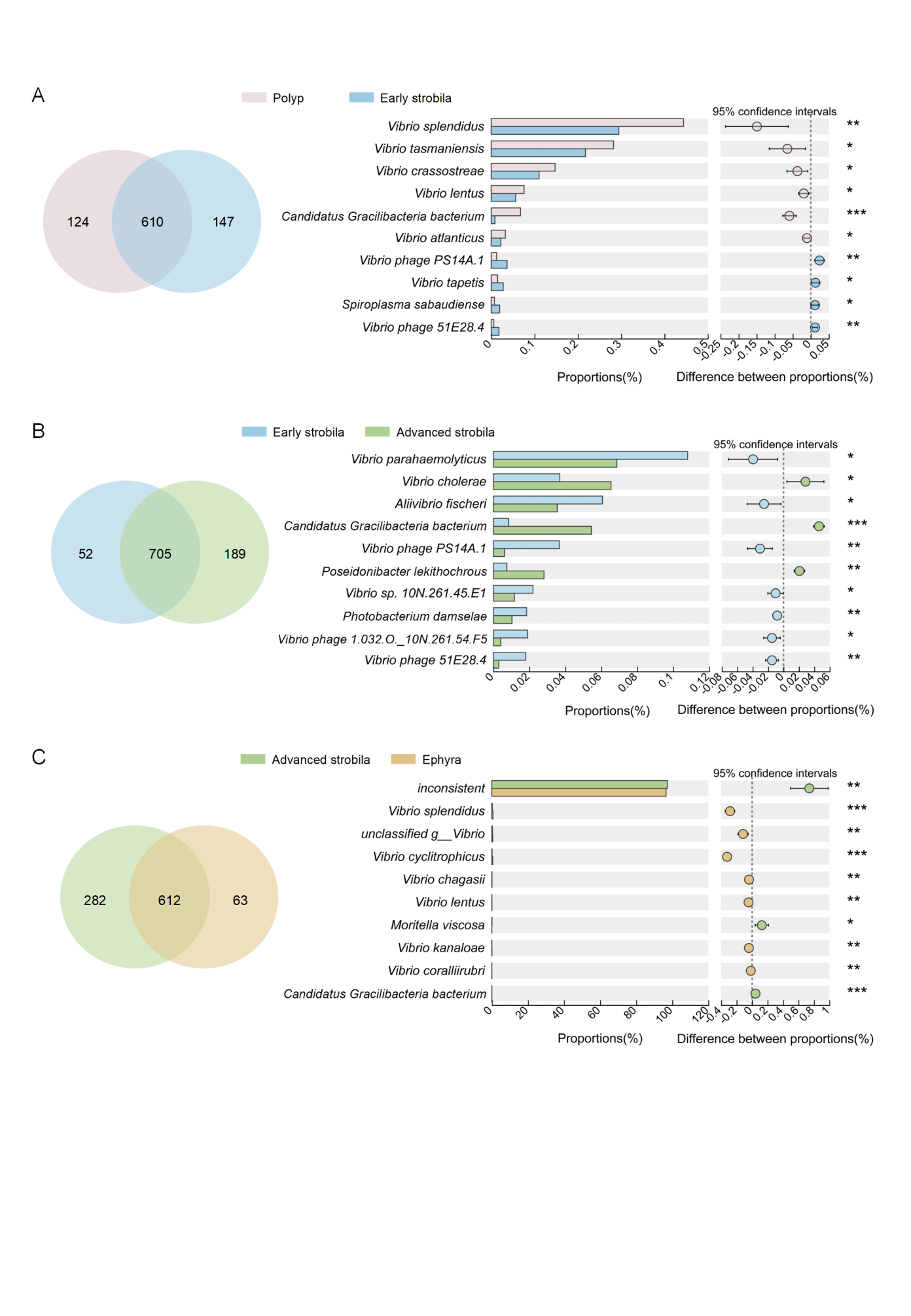


**Fig. S3 Microbial biomarkers between adjacent stages of *Aurelia coerulea*.** (A–C) Significantly differential species between polyp and early strobila stages, between early strobila and advanced strobila stages, and between advanced strobila and ephyra stages. The left Venn diagrams represent the number of shared and specific species between the two stages. The bar charts on the right represent the top 10 biomarkers with the highest abundance. Statistical analysis was perfomed using Student’s *t* test.


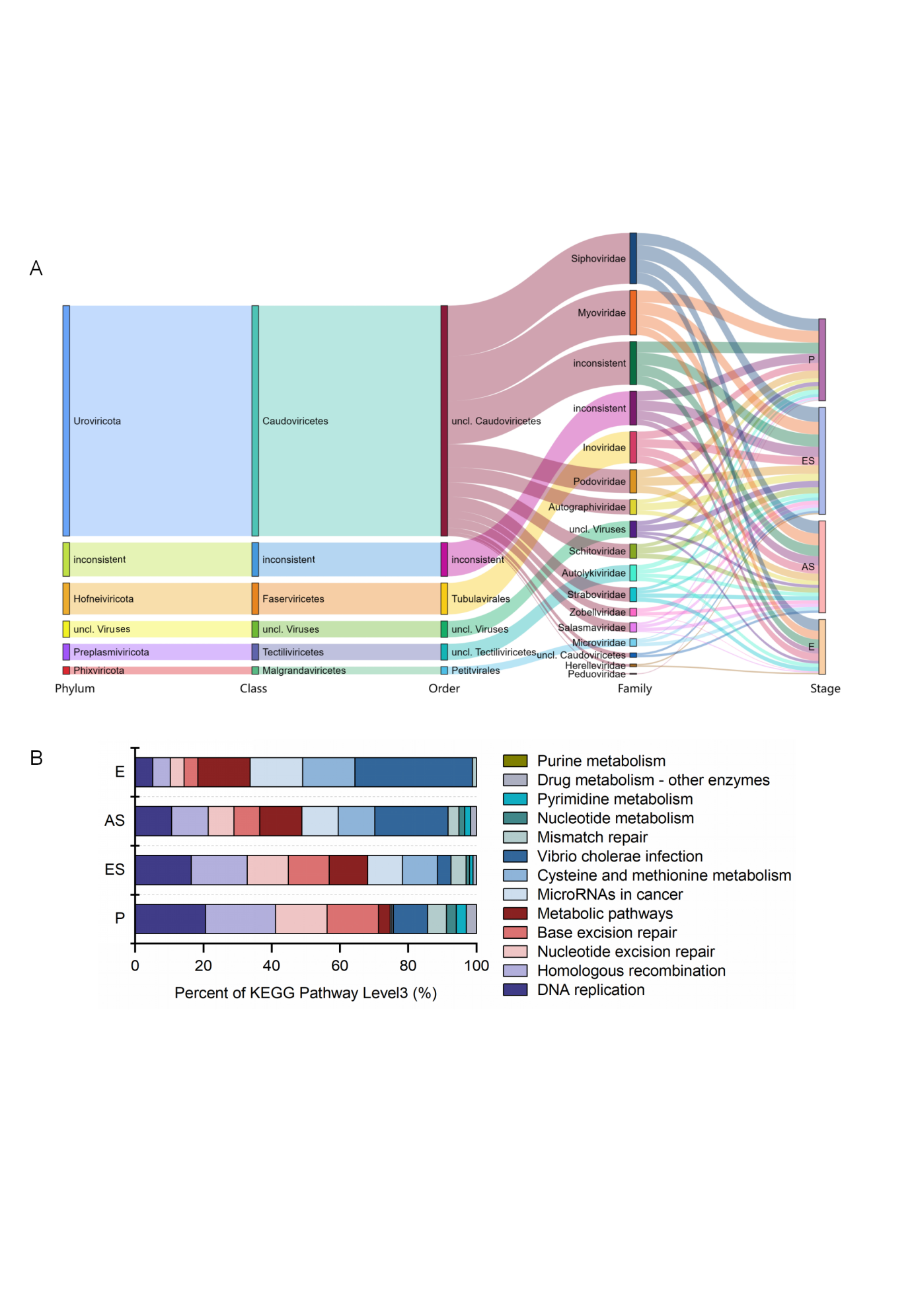


**Fig. S4 Taxonomic (A) and functional (B) composition of Viruses associated with *Aurelia coerulea*.** Taxonomic and functional abundance were calculated as the number of reads.


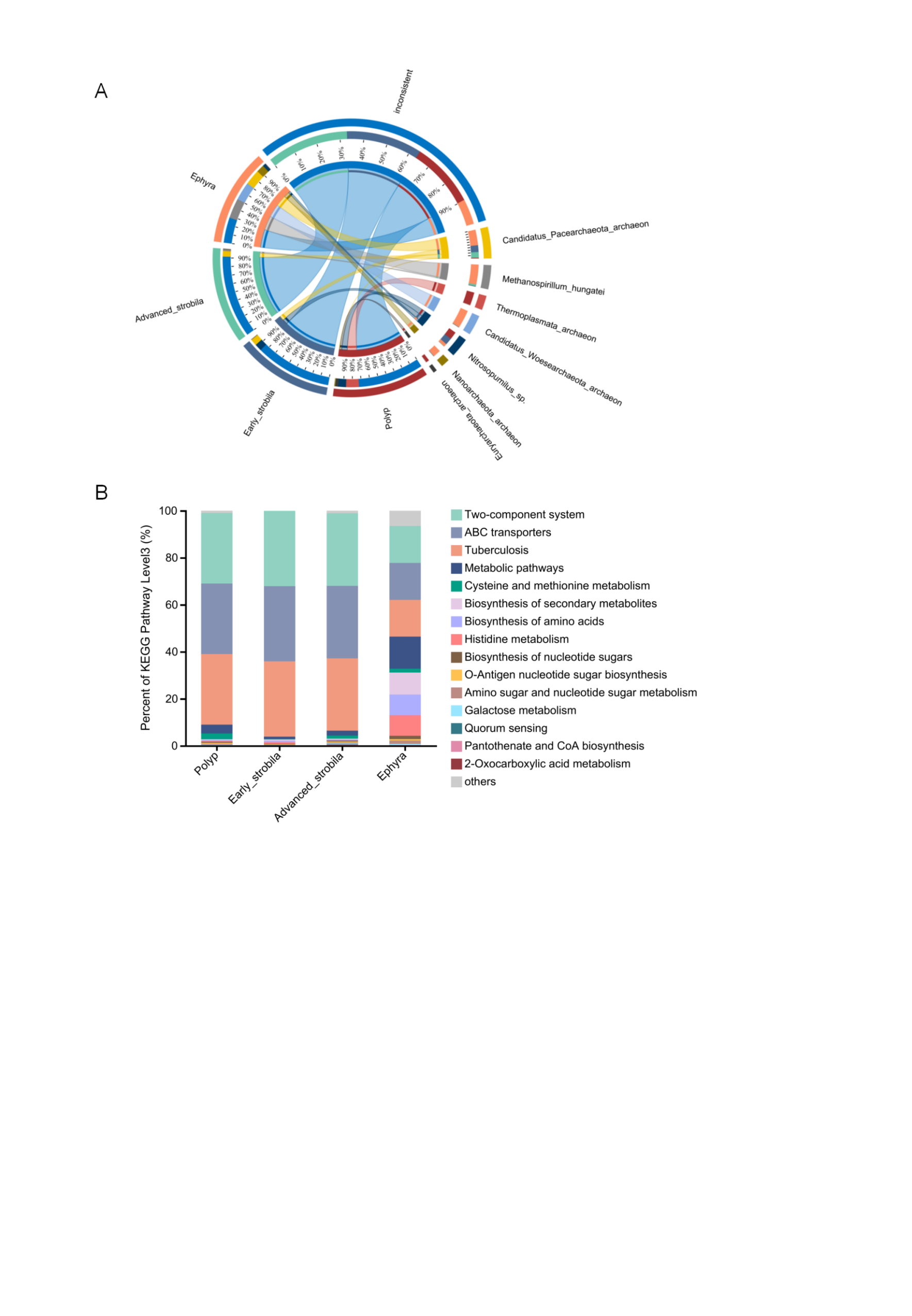


**Fig. S5 Taxonomic (A) and functional (B) composition of Archaea associated with *Aurelia*.** Taxonomic and functional abundance were calculated as the number of reads. The stacking bar plot of the functional composition only shows the top 15 KEGG pathways of Archaea.


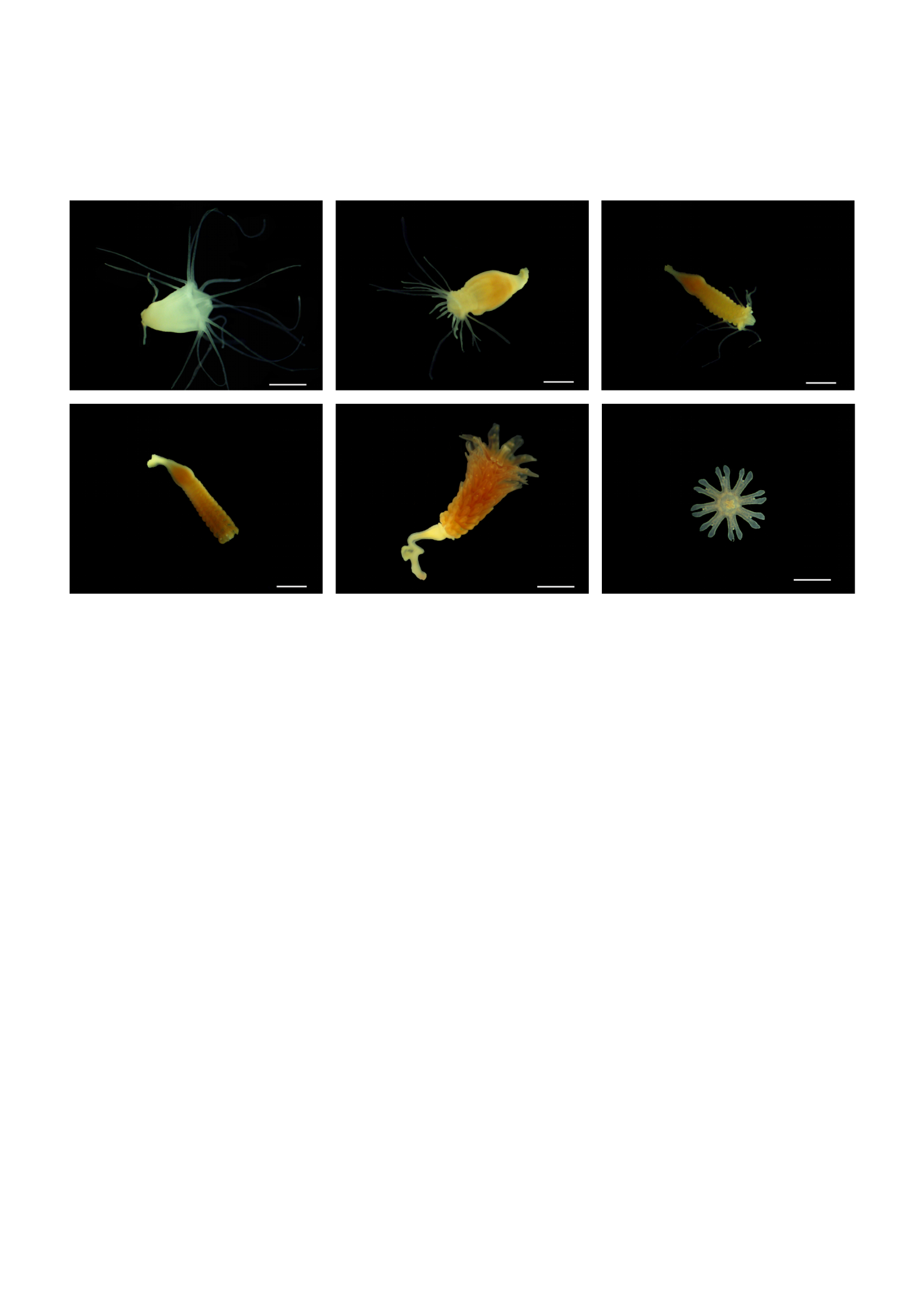


**Fig. S6 Photographs showing physiological changes during strobilation of *Aurelia coerulea*.** The scales bar shows 1 mm.


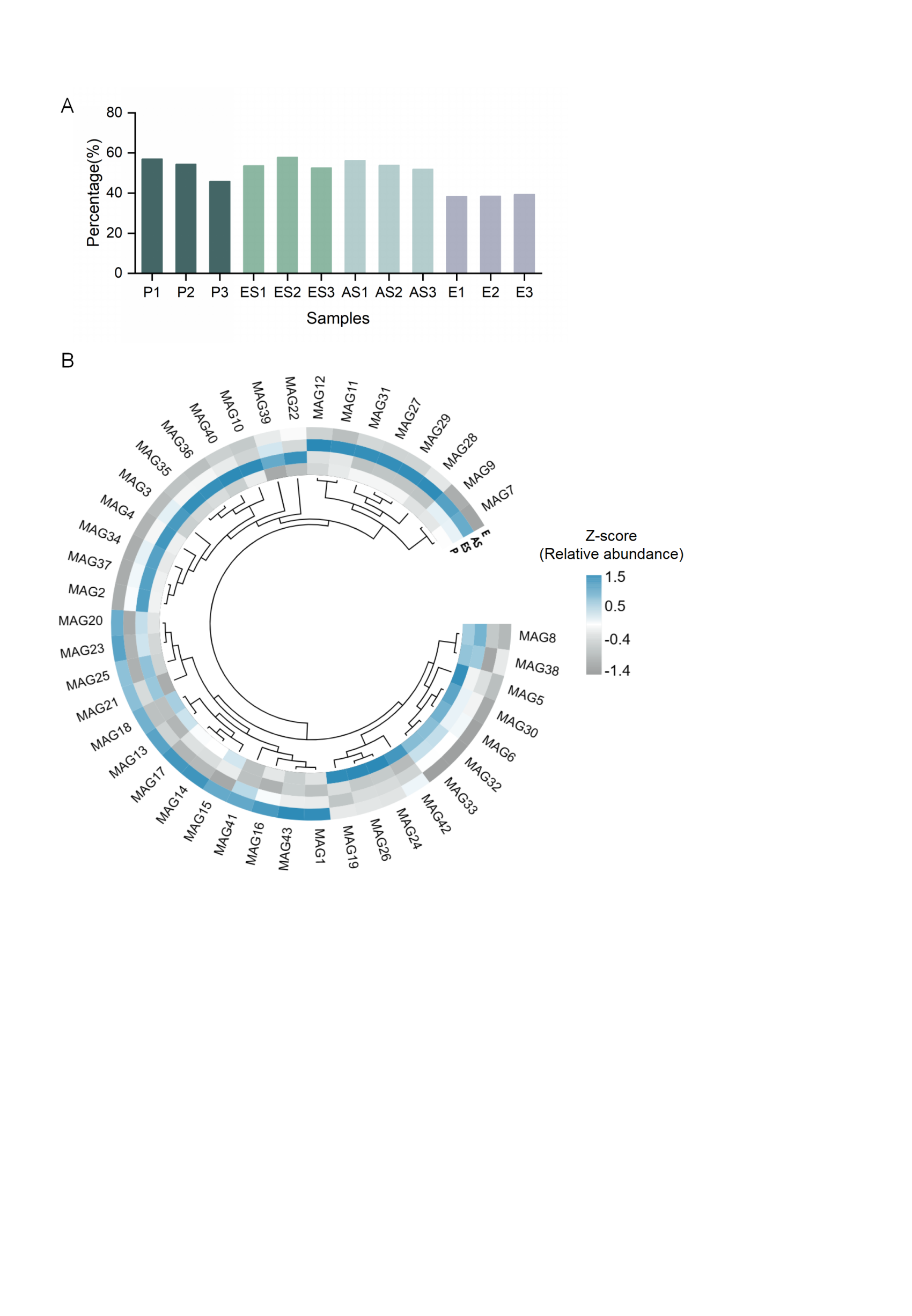


**Fig. S7 Proportion and abundance of 43 MAGs recovered from *Aurelia coerulea*.** (A) Percentage of recovered MAGs in the total community, determined using SingleM software. P1–P3, polyp stage replicates; ES1–ES3, early strobila stage replicates; AS1–AS3, advanced strobila stage replicates; E1–E3, ephyra stage replicates. (B) Relative abundance of MAGs during each life stage of *Aurelia*.
